# Early radiologic tumour volume response in non-metastatic rhabdomyosarcoma is not predictive for survival

**DOI:** 10.1007/s00247-025-06359-3

**Published:** 2025-08-14

**Authors:** Isabelle S.A. de Vries, Carlo Morosi, Gianni Bisogno, Veronique Minard-Colin, Beatrice Coppadoro, Ilaria Zanetti, Andrea Ferrari, Daniel Orbach, Salma Moalla, Myriam Ben-Arush, Christine Devalck, Roelof van Ewijk, Kieran McHugh, Meriel Jenney, Julia Chisholm, Gabriela Guillen, Henry Mandeville, Johannes H. M. Merks, Rick R. Van Rijn

**Affiliations:** 1 Princess Máxima Center for Pediatric Oncology, Heidelberglaan 25, 3584 CS Utrecht, Netherlands; 2 Department of Radiology, Fondazione IRCCS Istituto Nazionale dei Tumori, Milan, Italy; 3 Department of Women’s and Children’s Health, University of Padua, Padua, Italy; 4 Pediatric Hematology Oncology Division, University Hospital of Padua, Padua, Italy; 5 Department of Pediatric and Adolescent Oncology, Gustave-Roussy, Université Paris-Saclay, Villejuif, France; 6 Pediatric Oncology Unit, Fondazione IRCCS Istituto Nazionale dei Tumori, Milan, Italy; 7 SIREDO Oncology Center (Care, Innovation and Research for Children, Adolescents and Young Adults with Cancer), PSL University, Institut Curie, Paris, France; 8 Rambam Medical Center, Joan and Sanford Weill Pediatric Hematology Oncology and Bone Marrow Transplantation Division, Ruth Rappaport Children’s Hospital, Haifa, Israel; 9 Department Hematology Oncology, HUDERF, ULB, HUB, Brussels, Belgium; 10 Department of Radiology, Great Ormond Street Hospital for Children NHS Foundation Trust, London, United Kingdom; 11 Department of Paediatric Oncology, Children’s Hospital for Wales, Heath Park, Cardiff, United Kingdom; 12 Children and Young Peoples Unit, Royal Marsden Hospital and Institute of Cancer Research, Sutton, United Kingdom; 13 Department of Pediatric Surgery, Hospital Infantil Universitari Vall d’Hebron, Barcelona, Spain; 14 Department of Radiotherapy, Royal Marsden Hospital and The Institute of Cancer Research, Sutton, United Kingdom; 15 Division of Imaging and Oncology, University Medical Center Utrecht, University of Utrecht, Utrecht, Netherlands; 16 Department of Radiology and Nuclear Medicine, Amsterdam UMC – Emma Children’s Hospital, University of Amsterdam, Amsterdam, Netherlands

**Keywords:** Paediatric oncology, Radiological response assessment, Rhabdomyosarcoma, Tumour volume

## Abstract

**Background:**

There is an urgent need for early radiological markers predicting survival in paediatric rhabdomyosarcoma patients.

**Objective:**

To analyse the predictive value of early radiological tumour volume response to chemotherapy in non-metastatic rhabdomyosarcoma patients.

**Methods:**

The European paediatric Soft tissue sarcoma Study Group (E*p*SSG) RMS 2005 study prospectively included non-metastatic rhabdomyosarcoma patients. Patients with Intergroup Rhabdomyosarcoma Studies (IRS) postsurgical Group III (i.e. measurable disease) at diagnosis, with three tumour diameters available at diagnosis and following three cycles of chemotherapy, were included. Tumour response was categorized as complete response, partial response, minor partial response, stable disease, or progressive disease. Patients with progressive disease were excluded from analyses. Failure-free survival and overall survival were estimated with Kaplan-Meier using a landmark analysis. Prognostic factors were assessed using Cox proportional hazards analyses.

**Results:**

Of 1,674 patients aged < 18 years at diagnosis included in the E*p*SSG RMS 2005 study, 613 patients (median age 4.2 years, interquartile range 2.2–8.0, 346 males) were eligible for analysis. After three cycles of chemotherapy, 48 patients reached complete response, 371 partial response, 139 minor partial response, 48 stable disease, and seven patients progressive disease. Univariate Cox proportional hazards model showed no significant difference between response groups for failure-free survival or overall survival. In multivariate Cox regression analysis, tumour volume response was not an independent prognostic factor for failure-free survival or overall survival.

**Conclusion:**

Early radiological tumour volume response after the third cycle of neoadjuvant chemotherapy, with exclusion of progressive disease, does not predict survival in paediatric rhabdomyosarcoma patients.

**Graphical Abstract:**

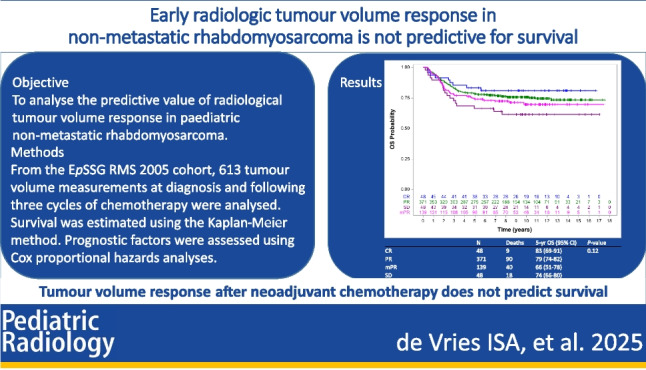

**Supplementary Information:**

The online version contains supplementary material available at 10.1007/s00247-025-06359-3.

## Introduction

In rhabdomyosarcoma, there is an ongoing search for early markers after initiation of therapy predicting patient outcome. First, to identify high-risk patients and thereby intensify or de-intensify treatment at an early stage. Second, to recognize effective new treatment strategies early on, and bring new promising treatments to phase III while at the same time stop trial-arms with unsuccessful regimens. Currently, the only valid endpoint for phase III trials available is survival, taking up to 5 years to 10 years to give results.

Historically, early radiologic tumour size response was widely used to decide whether to continue with standard chemotherapy or switch to second-line treatment in case of insufficient response. Furthermore, the efficacy of new treatment regimens was often evaluated based on radiologic tumour size response. In paediatric soft tissue sarcoma trials, response can be measured by two-dimensional (according to World Health Organization criteria) or three-dimensional (volumetric) measurements [1–5]. In the European *paediatric* Soft tissue sarcoma Study Group (E*p*SSG) RMS 2005 protocol, three-dimensional tumour measurements were used [6]. Most adult oncology trials use response assessments based on one-dimensional measurements determined by the Response Evaluation Criteria In Solid Tumors (RECIST 1.1) guidelines [7]. There is an ongoing debate if this approach should be used in paediatric tumours as well [8, 9].

The E*p*SSG RMS 2005 trial required at least 33% tumour reduction to continue first-line chemotherapy. Patients with volume response less than 33% were switched to second-line chemotherapy. However, existing literature on the predictive value of radiologic response is inconclusive, with some studies finding no evidence for a difference in survival based on radiological response [10–12], whereas others conclude that early radiological response is a predictive marker for survival [13–15].

Therefore, the purpose of this study was to analyse the predictive value of early tumour volume response prospectively collected in a large cohort of paediatric non-metastatic rhabdomyosarcoma patients with residual disease after initial surgery or biopsy, i.e. Intergroup Rhabdomyosarcoma Studies (IRS) postsurgical group III, included and treated according to the E*p*SSG RMS 2005 study protocol.

## Materials and methods

### Participant selection

Patients included in this post-hoc analysis of a prospective trial were treated in the E*p*SSG RMS 2005 study. E*p*SSG RMS 2005 was an international, prospective study (ClinicalTrials.gov: NCT00339118, registration date 19th of June 2006), including patients from 102 hospitals in 14 countries between October 1, 2005, and December 31, 2016. Patients from 0 to 25 years of age, with adequate cardiac, renal, and hepatic function with a pathologically proven, non-metastatic rhabdomyosarcoma were eligible for inclusion. Written informed consent was given by all patients and/or parents or both, according to the research ethics requirements of the individual institutions. Data collection was performed as previously published [6]. This cohort has been previously reported [6, 16–27].

Risk groups were identified based on six stratifying factors: histology, IRS postsurgical group, tumour site and size, node involvement, and patient age. Unfavourable risk factors were alveolar histology, a parameningeal, extremity, genitourinary bladder-prostate, or “other site” primary tumour location, 10 years and older, and a tumour size of 5 cm or larger. Based on these factors, treatment of patients was stratified according to their risk profile: low-risk, standard-risk, high-risk, and very high-risk group, each with a specific treatment strategy (Supplementary Material 1) [19, 20].

IRS group III patients (i.e. residual macroscopic disease following initial surgery or biopsy) were included in the standard-, high-, and very high-risk groups. For standard-risk patients, chemotherapy comprised four or five cycles of ifosfamide, vincristine, and actinomycin-D (IVA), dependent on risk subgroup and radiotherapy received, followed by either IVA or VA chemotherapy, nine cycles in total. High-risk patients were randomized to receive either four cycles of IVA or IVA plus doxorubicin (IVADo), followed by five cycles of IVA. Patients with very high-risk rhabdomyosarcoma received four cycles of IVADo followed by five cycles of IVA [6].

High-risk patients in clinical complete remission after nine cycles of neoadjuvant therapy were offered randomization between the addition of 6 months of maintenance therapy with low-dose vinorelbine/cyclophosphamide versus stopping treatment [16]. Very high-risk patients received IVADo/IVA and 6 months of maintenance chemotherapy (Supplementary Material 1) [19, 20].

The decision regarding local therapy, surgery and/or radiotherapy, was made based on risk group, tumour response after three cycles of neoadjuvant chemotherapy, and the possibility of resection of the remaining tumour without mutilation (delayed surgery).

Early tumour volume response after three cycles of neoadjuvant chemotherapy was assessed in IRS group III patients. According to the E*p*SSG RMS 2005 protocol guidelines, patients with insufficient tumour volume reduction at this timepoint, defined as less than 33% decrease in volume, were to be switched to second-line chemotherapy.

### Participants

From the EpSSG RMS 2005 study, all patients aged < 18 years at diagnosis with measurable disease at baseline, i.e. IRS group III, were included if three-dimensional tumour volume measurements were available for analysis both at baseline and directly after the third cycle of neoadjuvant chemotherapy. If measurements at one or both timepoints were missing, patients were excluded.

### Procedures

Response was assessed by comparing the initial tumour volume at diagnosis to the tumour volume at response evaluation. The tumour measurements were retrieved from the E*p*SSG RMS 2005 study database by the E*p*SSG International Data Centre. These measurements were prospectively collected by the radiologists at the local sites, either with MRI or CT. Tumour volume was calculated using: volume = π/6 × length × width × depth. The tumour volume response was assessed based on the volumes calculated from the actual tumour measurements reported in the E*p*SSG database from the radiology reports, instead of the reported resulting response group remotely entered into the E*p*SSG database by data managers. The tumour volume response was categorized according to the E*p*SSG RMS 2005 imaging protocol criteria (Supplementary Material 2).

### Outcomes

Failure-free survival and overall survival were defined as outcomes. Failure-free survival is the time from response assessment (landmark point) to disease progression, local and/or metastatic relapse, a second malignancy, or death from any cause. Overall survival is the time from response assessment to death from any cause.

### Statistical analysis

Associations between the groups and the characteristics were determined with Chi-square tests.

To calculate the 5-year failure-free survival and overall survival, a landmark analysis, with completion of the third cycle of neoadjuvant chemotherapy as the landmark, was performed with Kaplan–Meier estimators [28]. A log-rank test was used to compare the different response groups for failure-free survival and overall survival. The prognostic value of early radiologic response was evaluated in a univariate and multivariate analysis using Cox proportional hazards models. A stepwise selection was performed to select the significant variables in the multivariate analysis. Proportional hazards assumptions were verified for all the covariates by means of time-dependent variables and graphical methods evaluating the Schoenfeld residuals [29]. Patients with progressive disease were excluded from the Cox analyses and the calculation of Kaplan–Meier estimators due to small patient numbers (*n* = 7). Patients with missing data were excluded from analysis. *P*-values lower than 0.05 were considered statistically significant. All analyses were performed with SAS software, version 9.4 (SAS Institute, Cary, NC).

## Results

### Patient population

**Fig. 1:**
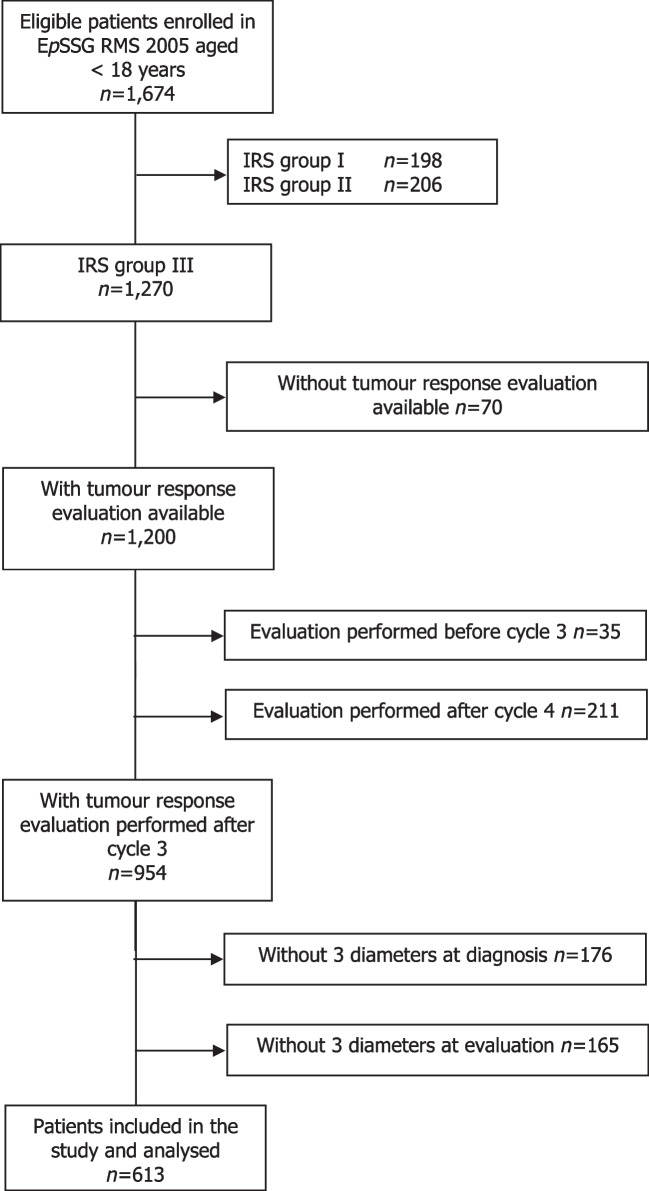
Consort diagram. *EpSSG RMS 2005* European *paediatric* Soft tissue sarcoma Study Group Rhabdomyosarcoma 2005, *IRS group* Intergroup Rhabdomyosarcoma Studies post-surgical group

In total, E*p*SSG RMS 2005 included 1,674 patients aged < 18 years at diagnosis. Of 1,270 IRS group III patients, 613 (48%) had three-dimensional measurements available at diagnosis and after three cycles of neoadjuvant chemotherapy and were included for this analysis (Fig. 1).

When comparing IRS group III patients included in the analysis to those excluded (i.e. without three-dimensional measurements available at any of the timepoints), patients with all measurements available after the third cycle were younger (*P* < 0.001), had more tumours at unfavourable sites (*P* < 0.001), and had larger tumours (*P* < 0.001) (Supplementary Material 3).

### Baseline characteristics

Of the 613 patients, 267 (44%) were female and the median age at diagnosis was 4.2 years (range 6 days – 17.9 years). Most patients, 426 of 613 patients (69%), had a tumour with embryonal histology; and, of the patients of whom the fusion status was known (82%), 398 of 505 patients (79%) had a tumour with negative *PAX-FOXO1* fusion status (Table 1).

**Table 1:** Clinical characteristics by response group based on volume reduction

	CR *n* (%) *n* = 48	PR *n* (%) *n* = 371	mPR *n* (%) *n* = 139	SD *n* (%) *n* = 48	PD *n* (%) *n* = 7	Total *n* (%) *n* = 613	*P-*value
**Age, years**							
≤ 1	5 (10.4)	21 (5.7)	5 (3.6)	12 (25.0)	5 (71.4)	48 (7.8)	** < 0.0001**
1–9	29 (60.4)	301 (81.1)	101 (72.7)	29 (60.4)	29 (60.4)	461 (75.2)	
10–17	14 (29.2)	49 (13.2)	33 (23.7)	7 (14.6)	14 (29.2)	104 (17.0)	
**Age, years**							
< 10	34 (70.8)	322 (86.8)	106 (76.3)	41 (85.4)	6 (85.7)	509 (83.0)	**0.0089**
≥ 10	14 (29.2)	49 (13.2)	33 (23.7)	7 (14.6)	1 (14.3)	104 (17.0)	
**Gender**							
Female	29 (60.4)	156 (42.0)	59 (42.5)	20 (41.7)	3 (42.9)	267 (43.6)	0.20
Male	19 (39.6)	215 (58.0)	80 (57.5)	28 (58.3)	4 (57.1)	346 (56.4)	
**Definitive histology**							
Alveolar RMS	16 (33.3)	100 (27.0)	26 (18.7)	11 (22.9)	-	153 (25.0)	**0.039**
Embryonal RMS	31 (64.6)	253 (68.2)	106 (76.3)	30 (62.5)	6 (85.7)	426 (69.5)	
Spindle cells/Leiom. RMS	-	13 (3.5)	7 (5.0)	6 (12.5)	1 (14.3)	27 (4.4)	
RMS NOS	1 (2.1)	5 (1.3)	-	1 (2.1)	-	7 (1.1)	
**Fusion status**							
Negative	28 (58.3)	233 (62.8)	102 (73.4)	30 (62.5)	5 (71.4)	398 (64.9)	0.12^a^
Positive	12 (25.0)	70 (18.9)	17 (12.2)	8 (16.7)	-	107 (17.5)	
Fusion status missing	8 (16.7)	68 (18.3)	20 (14.4)	10 (20.8)	2 (28.6)	108 (17.6)	
**Alveolar RMS (*n* = 153)**	**(*n* = 16)**	**(*n* = 100)**	**(*n* = 26)**	**(*n* = 11)**	**-**	**(*n* = 153)**	
Negative	2 (12.5)	25 (25.0)	6 (23.1)	2 (18.2)	-	35 (22.9)	0.78^a^
Positive	12 (75.0)	70 (70.0)	17 (65.4)	8 (72.7)	-	107 (69.9)	
Fusion status missing	2 (12.5)	5 (5.0)	3 (11.5)	1 (9.1)	-	11 (7.2)	
**Tumour primary site**							
Orbit	9 (18.8)	34 (9.2)	9 (6.5)	2 (4.2)	-	54 (8.8)	**0.0008**
HN no PM	9 (18.8)	33 (8.9)	7 (5.0)	2 (4.2)	1 (14.3)	52 (8.5)	
HN PM	13 (27.1)	91 (24.5)	48 (34.5)	13 (27.1)	-	165 (26.9)	
GU BP	4 (8.3)	69 (18.6)	22 (15.8)	11 (22.9)	1 (14.3)	107 (17.5)	
GU no BP	5 (10.4)	18 (4.9)	2 (1.4)	2 (4.2)	-	27 (4.4)	
Extremities	4 (8.3)	51 (13.8)	25 (18.0)	14 (29.2)	3 (42.9)	97 (15.8)	
Other sites	4 (8.3)	75 (20.2)	26 (18.7)	4 (8.3)	2 (28.6)	111 (18.1)	
**Tumour primary site**							
Favourable site	23 (47.9)	85 (22.9)	18 (13.0)	6 (12.5)	1 (14.3)	133 (21.7)	** < 0.0001**
Unfavourable site	25 (52.1)	286 (77.1)	121 (87.0)	42 (87.5)	6 (85.7)	480 (78.3)	
**Tumour size**							
a: ≤ 5 cm	32 (66.7)	132 (35.6)	36 (25.9)	18 (37.5)	4 (57.1)	222 (36.2)	** < 0.0001**
b: > 5 cm	16 (33.3)	239 (64.4)	103 (74.1)	30 (62.5)	3 (42.9)	391 (63.8)	
**T-invasiness**							
T1	27 (56.3)	159 (42.9)	60 (43.2)	29 (60.4)	4 (57.1)	279 (45.5)	0.072^b^
T2	21 (43.8)	208 (56.1)	79 (56.8)	19 (39.6)	2 (28.6)	329 (53.7)	
Tx	-	4 (1.1)	-	-	1 (14.3)	5 (0.8)	
**Loco-regional *N***							
N0	34 (70.8)	298 (80.3)	117 (84.2)	43 (89.6)	7 (100.0)	499 (81.4)	0.13^c^
N1	13 (27.1)	72 (19.4)	22 (15.8)	5 (10.4)	-	112 (18.3)	
Nx	1 (2.1)	1 (0.3)	-	-	-	2 (0.3)	
**Risk group**							
Standard risk	21 (43.7)	92 (24.8)	31 (22.3)	12 (25.0)	4 (57.1)	160 (26.1)	**0.0075**
High risk	19 (39.6)	244 (65.8)	99 (71.2)	33 (68.8)	3 (42.9)	398 (64.9)	
Very high risk	8 (16.7)	35 (9.4)	9 (6.5)	3 (6.3)	-	55 (9.0)	

*BP* bladder and prostate, *CR* complete response, *GU* genitourinary, *HN* head and neck, *mPR* minor partial response, *NOS* not otherwise specified, *PD* progressive disease, *PM* parameningeal *PR* partial response, *RMS* rhabdomyosarcoma, *SD* stable disease

^a^Patients with missing fusion status excluded

^b^Patients with Tx excluded

^c^Patients with Nx excluded

### Response assessment

At radiological tumour response evaluation after the third cycle of chemotherapy, 48 of 613 patients (7.8%) achieved complete response. Most patients, 371 patients (60.5%), had a partial response, 139 patients (22.7%) a minor partial response, 48 patients (7.8%) stable disease, and seven patients (1.1%) showed progressive disease. Supplementary Material 4 gives a detailed overview of the seven patients with progressive disease in this study. Younger age (*P* = 0.0089), tumour histology (*P* = 0.039), unfavourable tumour site (*P* < 0.001), large tumour size (*P* < 0.001), and clinical risk groups (*P* = 0.0075) were significantly associated with poorer tumour volume response (Table 1). *PAX-FOXO1*-fusion status, gender, tumour invasiveness, and loco-regional lymph node involvement were not significantly associated with any of the different response groups.

Furthermore, we observed that 90 of the 613 patients included (15%) scored a different response group when the tumour response was calculated based on the radiological measurements available, compared to the response group that was reported in the E*p*SSG central database, as is shown in Supplementary Material 5.

### Therapy

The E*p*SSG RMS 2005 protocol advised a switch to second-line chemotherapy when less than minor partial response was reached after three cycles of neoadjuvant chemotherapy. However, in 22 of 48 patients (46%) with stable disease and in one of seven patients (14%) with progressive disease, no changes in chemotherapy regimen were made after response evaluation. However, for the patients with calculated progressive disease, the reported response group within the E*p*SSG database was stable disease. Furthermore, two of 371 patients (< 1%) with partial response and five of 139 patients (4%) with minor partial response did switch to second-line chemotherapy, aiming to increase response to allow for the application of adequate local therapy.

Radiotherapy was performed in 35 of 48 patients (73%) with complete response, 304 of 371 patients (82%) with partial response, 117 of 139 patients (84%) with minor partial response, 34 of 48 patients (71%) with stable disease, and three of seven patients (43%) with progressive disease. Reasons not to perform radiotherapy were the decision of the centre (33%), a very young age (32%), early progressive disease (13%), parental refusal (6%), amputation before radiotherapy (3%), and optional according to risk group or site (13%).

Delayed surgery of primary tumour or lymph nodes was performed in five of 48 patients (10%) with complete response, 221 of 371 patients (60%) with partial response, 92 of 139 patients (66%) with minor partial response, 33 of 48 patients (69%) with stable disease, and two of seven patients (29%) with progressive disease. Of 353 patients who received delayed surgery, a complete resection (R0) could be achieved in 220 patients (62%). In patients with radiological complete response, this was achieved in five of five patients (100%), with partial response in 146 of 221 patients (66%), with minor partial response in 50 of 92 patients (54%), with stable disease in 18 of 33 patients (55%) and with progressive disease in one of two patients (50%).

Of all 613 patients, 267 patients (44%) received both radiotherapy and delayed surgery, 86 patients (14%) received delayed surgery only, 226 patients (37%) received radiotherapy only, and 34 patients (5%) received no local therapy.

### Survival

Overall, 219 of 613 patients (36%) experienced an event, and 158 (26%) died. Three hundred and ninety-four patients (64%) are alive in first complete response at last follow-up. The median follow-up time for alive patients was 117 months from the start of chemotherapy (interquartile range 90.0–144.7). Of the 219 patients who experienced an event, 92 patients (42%) had a local relapse, 12 (5%) had a regional lymph node relapse, nine (4%) had a lymph node and local relapse, 50 (23%) had a metastatic relapse with or without local or nodal relapse, 39 (18%) had progressive disease with or without nodal or metastatic relapse, 11 (5%) developed a second tumour, four (1%) died of toxicity (i.e. one of post-surgery septic shock, two of cardiac arrest in the context of a septic process, and one suspension of the protocol due to meningoencephalitis), and two (< 1%) died of other non-oncological reasons.

### Early response and effect on survival

**Fig. 2:**
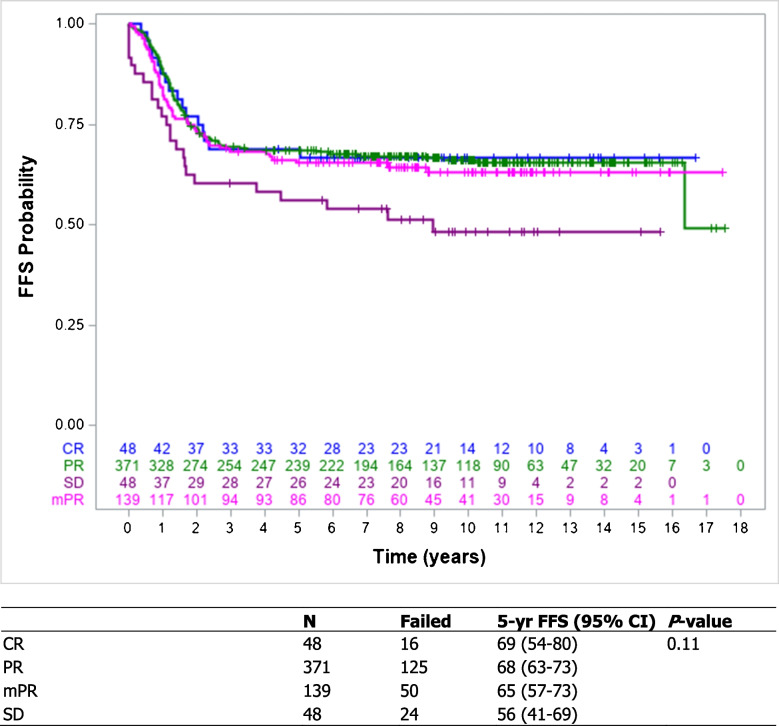
Kaplan–Meier estimates of failure-free survival based on early tumour volume response of 606 IRS group III patients with complete response, partial response, minor partial response, and stable disease included in the E*p*SSG RMS 2005 protocol. *5-yr* 5-year, *CI* confidence interval, *CR* complete response, *EpSSG* European *paediatric* Soft tissue sarcoma Study Group, *FFS* failure-free survival, *IRS* Intergroup Rhabdomyosarcoma Studies, *mPR* minor partial response, *PR* partial response, *SD* stable disease

**Fig. 3:**
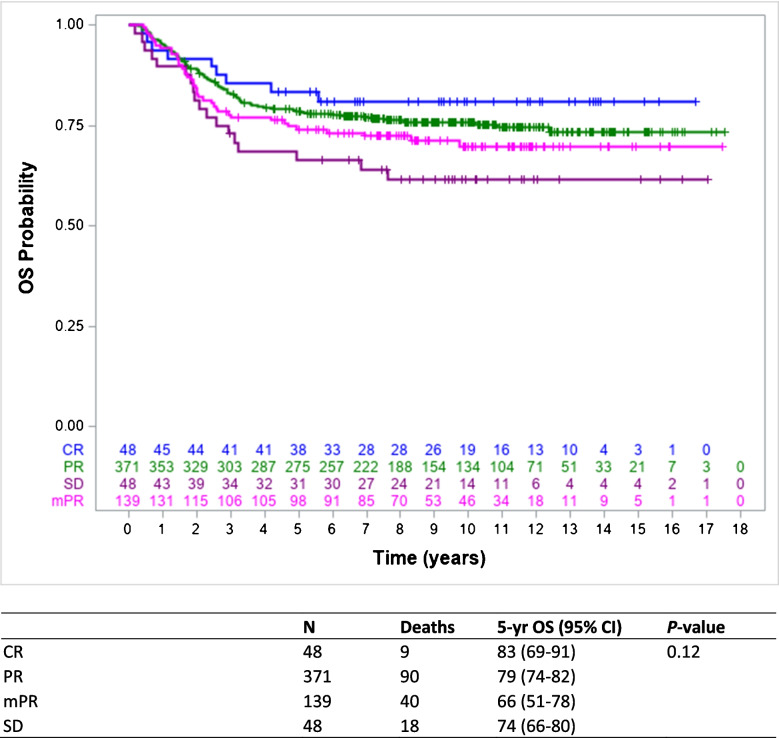
Kaplan–Meier estimates of overall survival based on early tumour volume response of 606 IRS group III patients with complete response, partial response, minor partial response, and stable disease included in the E*p*SSG RMS 2005 protocol. *5-yr* 5-year, *CI* confidence interval, *CR* complete response, *EpSSG* European *paediatric* Soft tissue sarcoma Study Group, *IRS* Intergroup Rhabdomyosarcoma Studies, *mPR* minor partial response, *OS* overall survival, *PR* partial response, *SD* stable disease

The estimated 5-year failure-free survival and overall survival for all 613 included patients were 66.5% (95% confidence interval (CI) 62.6%−70.1%) and 77.0% (95% CI 73.4%−80.1%), respectively. No significant differences were observed between the different response groups for failure-free survival or overall survival, *P* = 0.11 and *P* = 0.12, respectively (Figs. 2 and 3).

Univariate Cox proportional hazards model did not show a significant difference for failure-free survival and overall survival between the different response groups. Hazard ratios (HR) for partial response, minor partial response, and stable disease were 1.02 (95% CI 0.61–1.72), 1.13 (95% CI 0.64–1.98), and 1.75 (95% CI 0.93–3.29) (*P* = 0.11), respectively, for failure-free survival and 1.33 (95% CI 0.67–2.65), 1.63 (95% CI 0.79–3.37), and 2.23 (95% CI 1.00–4.96) (*P* = 0.13), respectively, for overall survival. The multivariate Cox regression analysis identified site (unfavourable; HR 1.49, 95% CI 1.04–2.13, *P* = 0.03), histology (unfavourable; HR 1.49, 95% CI 1.11–1.99, *P* = 0.01), and loco-regional lymph node involvement (N1; HR 1.47, 95% CI 1.07–2.03, *P* = 0.02) as independent prognostic factors for failure-free survival. For overall survival, age at diagnosis (≥ 10 years; HR 1.49, 95% CI 1.00–2.20, *P* = 0.05), site (unfavourable; HR 3.26, 95% CI 1.87–5.67, *P* < 0.001), and loco-regional lymph node involvement (N1; HR 2.33, 95% CI 1.66–3.26, *P* < 0.001) were identified as independent prognostic factors as well. For both failure-free survival and overall survival, tumour volume response was not identified as an independent prognostic factor.

## Discussion

Our study demonstrates that, in this large cohort of patients with IRS group III rhabdomyosarcoma, radiologic tumour volume response after three cycles of neoadjuvant chemotherapy, defined according to the E*p*SSG RMS 2005 response groups excluding progressive disease, does not predict failure-free survival or overall survival.

This result is comparable with the International Society of Pediatric Oncology (SIOP) Malignant Mesenchymal Tumor 95 (MMT-95) study cohort, and two Children’s Oncology Groups (COG) studies which did not find early radiological tumour size response prognostic for survival [10–12]. Similar to our study, these three studies excluded patients with progressive disease from analyses, as it is known that patients with early progressive disease have dismal prognosis [30].

On the contrary, the Cooperative Weichteilsarkom Studiengruppe (CWS) did include patients with progressive disease in their analysis and found early radiological response to be a prognostic factor for survival [14]. The same is demonstrated by an Italian single-centre study where a multivariable Cox model showed that radiological tumour size response was predictive for overall survival. Again, patients with progressive disease were included in this analysis [15].

As described in the systematic review of van Ewijk et al., the latter two studies underline the poor prognosis of patients with progressive disease, but the significance of early radiological tumour response in patients without progressive disease is indistinct, as the larger group without progressive disease was not analysed separately [31].

Several limitations are important to address when interpreting the observed results. First, due to the post-hoc nature of our study, more than half of the IRS group III patients were excluded for not having tumour response evaluation available directly after the third cycle of neoadjuvant chemotherapy or not having all three-dimensional measurements available at either baseline or at response evaluation (Fig. 1).

Second, in the E*p*SSG RMS 2005 study, all response measurements were performed by local radiologists, with the potential for interobserver variability that may influence the results [32]. A study performed in 124 consecutive patients treated in two referral centres showed that a considerable number of patients, up to 14% (95% CI 6—23%), could have been treated differently due to interobserver variability when scored independently by experienced radiologists according to predefined guidelines [33]. And although the E*p*SSG RMS 2005 trial database did allow for upload of radiology reports, a standardized radiology report was not in place; we advocate a standardized radiology report for future studies, with an explicit conclusion, including all necessary tumour measurements and the percentage of volume reduction to ensure homogenous radiological assessments [34]. For the current European overarching Frontline and Relapse Rhabdomyosarcoma (FaR-RMS) study (ClinicalTrials.gov: NCT04625907) there are detailed imaging guidelines in place, including protocols to be used for the different imaging modalities to harmonize imaging. In addition, there is a specific Frontline and Relapse Rhabdomyosarcoma study imaging repository to allow for central review [35].

Third, the E*p*SSG RMS 2005 protocol specified a chemotherapy switch for patients with stable disease and progressive disease, which could introduce potential bias due to the change in therapy they received. However, randomized controlled studies have shown that intensification of the current IVA therapy does not translate into a survival advantage [1, 6]. Thus, it is assumed that patients with stable disease have no improved survival on second-line chemotherapy.

Lastly, the ideal timing for early response evaluation in patients with rhabdomyosarcoma is not known. The current standard is to evaluate after the third cycle of chemotherapy, which is dictated by the time point to plan local therapy.

This study reports on the analyses of radiological volume response in the largest cohort of non-metastatic RMS patients prospectively treated according to contemporary guidelines within the framework of the most recent European study, with a long period of follow-up, and three tumour dimensions available at diagnosis and after three cycles of chemotherapy.

The results of this study support the current treatment guidelines, in which IRS group III patients who achieve complete response, partial response, minor partial response, or stable disease after three cycles of neoadjuvant chemotherapy remain on standard first-line chemotherapy.

Moreover, these results suggest that early radiological tumour volume response, defined according to the E*p*SSG RMS 2005 response groups, is not a predictive biomarker to be utilized in clinical trials. Additional imaging measures, like the peak standardized uptake value (SUVpeak) in [18F]FDG-PET or the apparent diffusion coefficient (ACD) in diffusion-weighted MRI, should be explored to find validated, early surrogate biomarkers of long-term survival. Evidence for both modalities in initial staging and response to treatment modalities in rhabdomyosarcoma patients is still limited [36–38]. However, both quantitative markers have been shown to be predictive markers for response in other solid tumours [39, 40]. Imaging studies within the current FaR-RMS study prospectively study SUVpeak and ADC response as predictive biomarkers for survival.

## Conclusion

This study confirms that early radiological tumour volume response, defined according to the E*p*SSG RMS 2005 response groups with exclusion of progressive disease, does not predict failure-free survival or overall survival in IRS group III patients and should not be considered an early surrogate endpoint in paediatric rhabdomyosarcoma.

## Supplementary Information

Below is the link to the electronic supplementary material.

Supplementary file1 (DOCX 170 KB)

## Data Availability

All data in this manuscript are part of the EpSSG RMS 2005 repository, which is under the management of the EpSSG International Data Centre.
