# Supplementary material for: Early radiologic tumour volume response in non-metastatic rhabdomyosarcoma is not predictive for survival
Source: Pediatr Radiol. 2025 Aug 14;55(10):2160–70. doi: 10.1007/s00247-025-06359-3 (PMC12513956; doi:10.1007/s00247-025-06359-3)
Supplement: Supplementary file 1 — Supplementary file1 (DOCX 170 KB) [file 247_2025_6359_MOESM1_ESM.docx]

**Supplemental data**

**Supplementary Material 1.** E*p*SSG RMS 2005 study risk group stratification and therapy as presented by Glosli et al.[19]
*EpSSG RMS 2005* European *paediatric* Soft tissue sarcoma Study Group RMS 2005, *IRS* Intergroup Rhabdomyosarcoma Studies, *IVA* ifosfamide, vincristine and actinomycin-D, *IVADo* ifosfamide, vincristine, actinomycin-D and high dose doxorubicin, *VA* vincristine and actinomycin-D.


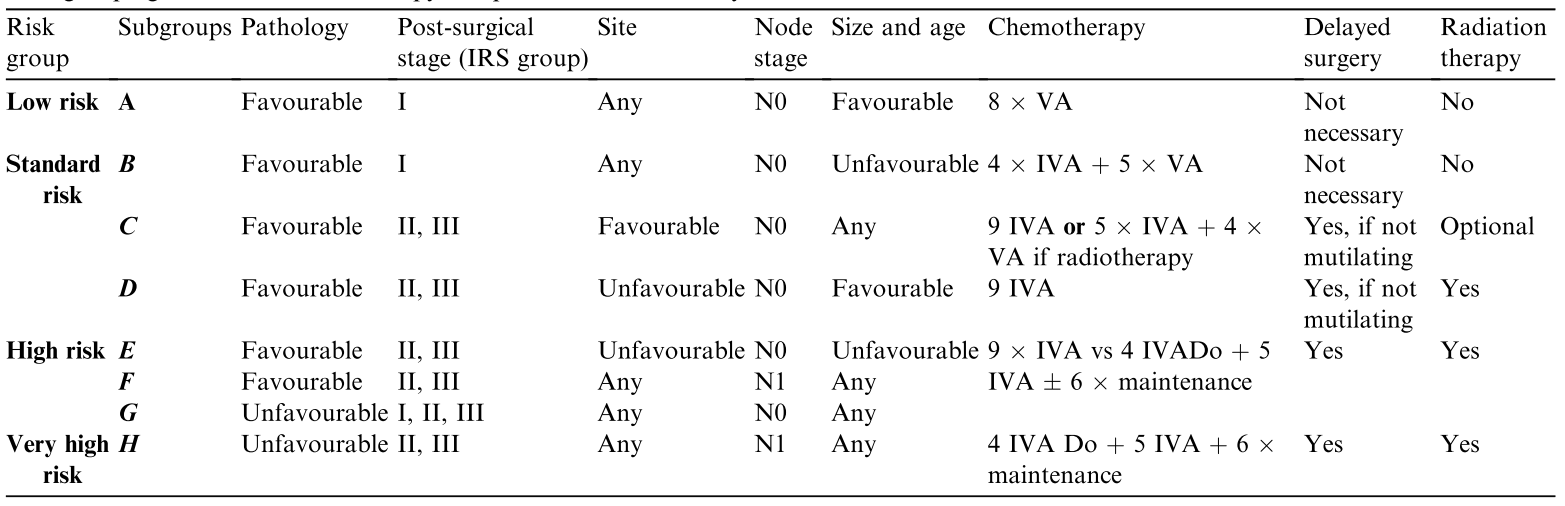


**Supplementary Material 2**. E*p*SSG RMS 2005 tumour volume response groups based on tumour volume response of increase.

*EpSSG RMS 2005* European *paediatric* Soft tissue sarcoma Study Group RMS 2005.

|  | |
| --- | --- |
| Complete response | Complete disappearance of all visible disease |
| Partial response | Tumour volume reduction ≥ 66% but < 100% |
| Minor partial response | Tumour volume reduction ≥ 33% but < 66% |
| Stable disease | Tumour volume reduction <33% and increase <40% |
| Progressive disease | Any increase in volume ≥ 40%, or appearance of new lesions |

**Supplementary Material 3.** Clinical characteristics of IRS group III patients excluded (with less than 3 measurements available both at diagnosis and after 3 cycles of chemotherapy) and included (with 3 measurements available at both timepoints)**.**^a^ Patients with missing fusion status excluded. ^b^ Patients with size x excluded. ^c^ Patients with Tx excluded.
^d^ Patients with Nx excluded.
*BP* bladder and prostate, *GU* genitourinary, *HN* head and neck, *NOS* not otherwise specified, *PM* parameningeal, *RMS* rhabdomyosarcoma.

|  | **Less than**  **3 measurements**  ***n* (%)**  ***n*=341** | **3 measurements**  ***n* (%)**  ***n*=613** | **Total**  ***n* (%)**  ***n*=954** | ***P*-value** |
| --- | --- | --- | --- | --- |
| Age, years |  |  |  |  |
| ≤ 1 | 18 (5.3) | 48 (7.8) | 66 (6.9) | **0.0002** |
| 1-9 | 227 (66.6) | 461 (75.2) | 688 (72.1) |  |
| 10-17 | 96 (28.2) | 104 (17.0) | 200 (21.0) |  |
| **Gender** |  |  |  |  |
| Female | 149 (43.7) | 267 (43.6) | 416 (43.6) | 0.97 |
| Male | 192 (56.3) | 346 (56.4) | 538 (56.4) |  |
| **Histology** |  |  |  |  |
| Favourable RMS | 239 (70.1) | 453 (73.9) | 692 (72.5) | 0.21 |
| Unfavorable RMS | 102 (29.9) | 160 (26.1) | 262 (27.5) |  |
| **Fusion status** |  |  |  |  |
| Negative | 195 (57.2) | 398 (64.9) | 593 (62.2) | 0.23 |
| Positive | 65 (19.1) | 107 (17.5) | 172 (18.0) |  |
| Fusion status missing | 81 (23.8) | 108 (17.6) | 189 (19.8) |  |
| **Tumour primary site** |  |  |  |  |
| Orbit | 48 (14.1) | 54 (8.8) | 102 (10.7) | **<0.0001** |
| HN no PM | 39 (11.4) | 52 (8.5) | 91 (9.5) |  |
| HN PM | 139 (40.8) | 165 (26.9) | 304 (31.9) |  |
| GU BP | 37 (10.9) | 107 (17.5) | 144 (15.1) |  |
| GU no BP | 18 (5.3) | 27 (4.4) | 45 (4.7) |  |
| Extremities | 26 (7.6) | 97 (15.8) | 123 (12.9) |  |
| Other sites | 34 (10.0) | 111 (18.1) | 145 (15.2) |  |
| **Tumour primary site** |  |  |  |  |
| Favourable site | 105 (30.8) | 133 (21.7) | 238 (25.0) | **0.0019** |
| Unfavorable site | 236 (69.2) | 480 (78.3) | 716 (75.0) |  |
| **Tumour size** |  |  |  |  |
| a: ≤ 5 cm | 162 (48.5) | 222 (36.2) | 384 (40.6) | **0.0002 ^b^** |
| b: > 5 cm | 172 (51.5) | 391 (63.8) | 563 (59.4) |  |
| **T-invasiness** |  |  |  |  |
| T1 | 152 (44.6) | 279 (45.5) | 431 (45.2) | 0.76 ^c^ |
| T2 | 187 (54.8) | 329 (53.7) | 516 (54.1) |  |
| T0/Tx | 2 (0.6) | 5 (0.8) | 7 (0.7) |  |
| **Loco-regional N** |  |  |  |  |
| N0 | 263 (77.1) | 499 (81.4) | 762 (79.9) | 0.13 ^d^ |
| N1 | 76 (22.3) | 112 (18.3) | 188 (19.7) |  |
| Nx | 2 (0.6) | 2 (0.3) | 4 (0.4) |  |

**Supplementary Material 4.** Overview of patients with progressive disease.
*Carbo* carboplatin, *Cylco* cyclophosphomide, *Doxo* doxorubicin, *GUPB* genitourinary bladder prostate, *HNnPM* head and neck non-parameningeal, *Ir* irinotecan, *IVA* ifosfamide, vincristine and actinomycin-D, *IVADo* ifosfamide, vincristine, actinomycin-D and high dose doxorubicin, *mPR* minor partial response, *PD* progressive disease, *Response RDE* response as entered by participating centres in the remote data entry platform, *RT* radiotherapy, *Sec. Surg.* secondary surgery, *SD* stable disease, *Topo* topotecan, *VA* vincristine and actinomycin-D, *VAC* vincristine, actinomycin-D and cyclophosphamide, *VIT* vincristine, irinotecan and temozolomide, *VP16* etoposide, *Yrs* years.

| **Response**  **group** | Response  RDE | Volume growth (%) | Site | Age  (yrs) | Type of event | Time from diagnosis to event  (months) | RT | Sec.  Surg. | Chemotherapy before evaluation | Chemotherapy after evaluation |
| --- | --- | --- | --- | --- | --- | --- | --- | --- | --- | --- |
| PD | mPR | 42% | Other | 4.8 | No event | - | Yes | No | 3 IVA | Topo/Carbo + Doxo/Cyclo + VP16 |
| PD | SD | 364% | Limbs | 0.4 | No event | - | Yes | Surg PT R0 | 3 IVA | Topo/Cyclo + Topo/Carbo |
| PD | SD | 79% | Limbs | 15.2 | No event | - | Yes | Surg PT R1 | 5 IVA | Stop chemotherapy |
| PD | PD | 194% | Other | 0.3 | PD | 2.4 | No | No | 3 IVA | Topo/VCR/Doxo and Topo/Carbo |
| PD | PD | 329% | GUBP | 0.2 | PD | 2.2 | No | No | 3 IVA | Doxo/Carbo + VP16 + VIT |
| PD | PD | 111% | HNnPM | 0.0 | PD | 2.5 | No | No | 1 VA+1 VAC | 1 IVADo |
| PD | PD | 438% | Limbs | 0.1 | PD | 2.5 | No | No | 4 IVA | Doxo/Carbo/Cyclo |

**Supplementary Material 5.** Overview of calculated volume response groups based on the measurements in the E*p*SSG central database and the reported response group that was entered by the local data manager in the remote data entry database.
*CR* complete response, *EpSSG* European *paediatric* Soft tissue sarcoma Study Group, *mPR* minor partial response, *PD* progressive disease, *PR* partial response, *RDE* remote data entry, *SD* stable disease, *VGPR* very good partial response.

| **Based on volume reduction** | **Stated in the RDE System** | | | | | | **Total**  ***n* (%)**  ***n*=613** |
| --- | --- | --- | --- | --- | --- | --- | --- |
|  | CR  *n*=46 | VGPR  *n*=104 | PR  *n*=284 | mPR  *n*=123 | SD  *n*=47 | PD  *n*=9 |  |
| CR | 45 | 3 | - | - | - | - | 48 (7.8) |
| PR | 1 | 99 | 248 | 19 | 4 | - | 371 (60.5) |
| mPR | - | 2 | 35 | 94 | 8 | - | 139 (22.7) |
| SD | - | - | 1 | 9 | 33 | 5 | 48 (7.8) |
| PD | - | - | - | 1 | 2 | 4 | 7 (1.1) |
